# Association between Maternal and Fetal Genetic Variants and Preeclampsia: Evidence from a Meta-Analysis

**DOI:** 10.3390/cimb46080489

**Published:** 2024-08-01

**Authors:** Tung Nguyen-Thanh, Phuong-Thao Nguyen-Vu, Quy-Anh Le-Thi, Thao-Nguyen Phan-Thi, Thi-Minh-Thi Ha

**Affiliations:** 1 Regenerative Medicine Core Research Group, Faculty of Basic Science, University of Medicine and Pharmacy, Hue University, 6 Ngo Quyen Street, Hue 49000, Vietnam; 2 Department of Biotechnology, University of Verona, 37134 Verona, Italy; 3 Institute of Biomedicine, University of Medicine and Pharmacy, Hue University, 6 Ngo Quyen Street, Hue 49000, Vietnam; 4 Department of Medical Genetics, University of Medicine and Pharmacy, Hue University, Hue 49000, Vietnam

**Keywords:** preeclampsia, maternal and fetal polymorphism, genetic variants, FLT1, ACE

## Abstract

The objective of this meta-analysis was to evaluate the association between maternal and fetal genetic variants and the risk of preeclampsia, a pregnancy-related condition that affects women. Despite the unclear role of these genetic factors in the development of preeclampsia, this analysis aimed to provide insights into the potential contributing factors. An electronic search of online databases was conducted to identify relevant studies. Stata SE software was used for the meta-analysis. A random-effects model was used to establish the association between the genetic variants and preeclampsia risk. Egger’s test was utilized to evaluate publication bias. Ten observational studies were selected from databases that met the inclusion criteria and included seven genes and twenty polymorphisms to analyze preeclampsia susceptibility influenced by the genetic background of both the mother and fetus. Our meta-analysis revealed that both the maternal and fetal polymorphisms, FLT1 rs4769613, were significantly associated with the risk of preeclampsia. However, the association between the maternal ACE rs4646994 polymorphism and preeclampsia risk was not statistically significant. Nevertheless, a significant association was observed between the fetal ACE rs4646994 polymorphism and preeclampsia in a dominant genetic model. In this study, the associations between maternal and fetal polymorphisms in ERAP2, VEGF, VDR, REN, and MMP were not statistically significant. According to the available evidence, maternal and fetal polymorphisms can impact the likelihood of developing preeclampsia. Additional research is required to fully understand the underlying mechanisms connecting maternal and fetal polymorphisms to preeclampsia, and to formulate recommendations for screening pregnant women based on these genetic variations.

## 1. Introduction

Preeclampsia (PE), an obstetric complication characterized by new-onset hypertension, frequently manifests after the 20th week of gestation [1]. It represents the most severe pregnancy complication, resulting in cesarean section, prematurity, chronic heart failure, and/or maternal kidney failure [2,3]. PE is usually categorized into two types according to the gestational age at which it initiates: Type I PE, or placental PE, which starts before 34 weeks of gestation, and Type II PE, or maternal PE, which begins after 34 weeks of gestation. Type I PE, caused by abnormal placental development in early gestation, progresses rapidly and can result in fetal growth restriction, leading to various complications later in life [4,5,6].

Several studies have reported that preeclampsia (PE) is a complex disorder that arises from the interplay between genetic, immunological, and environmental factors. This condition can be attributed to abnormal changes in the remodeling of spiral arteries, placental ischemia, and oxidative stress and progress in two stages. The first stage involves abnormal placentation, while the second stage involves dysregulation of anti-angiogenic factors and angiogenic imbalance, ultimately leading to PE clinical syndrome during the second and third trimesters of pregnancy [2,4,5,7].

The pathogenesis of preeclampsia (PE) remains unclear; however, the placenta is believed to be at the center of the etiology map [2,5,8,9,10]. Researchers have highlighted sFLT1 in the pathogenesis of preeclampsia and its role in the clinical syndrome of PE owing to its high protein levels of sFLT1 in maternal blood experiments and high sFLT1 mRNA expression in preeclamptic placentas [2,5]. At the molecular level, miRNAs are thought to be associated with the mechanisms and prediction of PE [8,9,11]. PE is likely an immune maladaptation that causes malnormal placentation by disrupting the establishment of immune tolerance, including TNFα, IFN-γ, IL-1, IL-4, IL-10, IL-27, HLA-G, TGF-β, E, HLA-C2, and KIR [8]. Molecular pathways that participate in the pathology of PE include the prolactin signaling pathway, peptide hormone metabolism, glycoprotein hormones, hormone ligand-binding receptors, peptide hormone biosynthesis, steroid hormone metabolism, steroid synthesis, the AMP-activated protein kinase (AMPK) signaling pathway, and the FoxO signaling pathway [11,12,13,14,15].

Preeclampsia is strongly associated with various molecular pathways. Researchers worldwide have conducted numerous studies on single-nucleotide polymorphisms (SNPs) to understand the genetic basis of preeclampsia. These studies have focused on genetic variations in anti-inflammatory mediators, vascular- and angiogenesis-related genes, histocompatibility-related genes, genes involved in metabolic changes during pregnancy, detoxification, DNA repair, and apoptosis [16,17]. Various SNPs have been associated with preeclampsia, including *FLT1* rs4769613 [8], *VEGF* rs3025039, and rs2010963 [18,19], *VDR* rs1544410 polymorphism [20,21], *ERAPT1* rs30187, [8,22], *TGF-β1* rs1800469 [23], prothrombin G20210A SNP [24], *SOD2 A16V* polymorphism [25], *eNOS* c.894 T [26], *XPC* rs2228000 [27], and the C1431T variant of *PPARγ* [28]. However, some SNPs that have been explored may not be associated with preeclampsia risk, such as TLR4 rs4986790 and rs4986791 [29] or ROS1 rs9489124 [30].

Scientists have increasingly focused on the association between maternal, fetal, and paternal gene variants, especially SNPs, as well as the development of preeclampsia (PE). However, several studies have found no evidence to support the notion that SNPs play a role in PE [31,32,33,34]. Additionally, there have been no reports linking GSTP1 Val105, eNOS 298Asp, and LPL -93G polymorphisms to the three genotypes of mothers, fathers, and neonates with PE [35]. Maternal and offspring VEGF polymorphisms can increase the risk of maternal PE in Romanian and Han Chinese pregnant women through the VEGF-T936 allele and VEGF-A rs2010963 polymorphism [36,37]. The placental FLT1 rs4769613 C allele was identified as a risk factor in two studies based on GWAS data [38,39], and sFlt-1 is a soluble splice variant of the full-length membrane receptor VEGFR-1.

Prior investigations have been constrained to exploring the association between genetic variations in mothers and preeclampsia. No meta-analysis has assessed the association between genetic variations in offspring and the occurrence of preeclampsia. The current study was conducted to examine the association between genetic alterations in pregnant women and their children and the initiation of preeclampsia. It has been postulated that genetic variations in children could influence the occurrence of maternal preeclampsia.

## 2. Materials and Methods

### 2.1. Search Strategy and Identification of Relevant Studies

A thorough exploration was carried out utilizing computer-based methods across a variety of online databases, including PubMed, Embase, Web of Science, and Google Scholar, to pinpoint pertinent research. The search criteria encompassed terms such as ‘Preeclampsia’ or ‘Pre-eclampsia’ and ‘Polymorphisms’ or ‘Genetic Polymorphism’ or ‘Single nucleotide polymorphism’ or ‘SNP’ or ‘variant’ or ‘genotype’ or ‘mutation’. Subsequently, the identified articles underwent individual scrutiny, with relevant research chosen for inclusion in the meta-analysis.

### 2.2. Criteria for Inclusion and Exclusion

Studies were selected based on the following criteria: (a) they examined the association between genetic variations and susceptibility to PE; (b) they included the genotype and allele frequencies of both cases and controls for the analyzed polymorphisms; (c) they presented original data; (d) they employed a case–control, cohort, or cross-sectional study design; and (e) they were published in English. Studies were excluded if they (a) used a different study design than case–control, cohort, or cross-sectional; (b) did not contain original data or were not relevant to the current analysis; and (c) lacked genotypic distribution or allele frequency data.

### 2.3. Data Extraction 

The extracted data elements included the first author’s name, publication year, ethnicity, genotyping method, single-nucleotide polymorphism (SNP), early- or late-onset stratification groups, number of case and control samples, maternal and fetal genotype distribution, and maternal and fetal allele frequencies in cases and controls. The genotype distribution of each gene was assessed for compliance with the Hardy–Weinberg equilibrium. The data extraction process was carried out separately by three authors (PTNV, QALT, and TNPT) and subsequently validated by the corresponding author.

### 2.4. Risk of Bias Assessment

The quality of the inclusion of trials was assessed using the Risk of Bias Assessment tool for Non-randomized Studies (RoBANS) [40]. Two authors (PTNV, QALT) independently assessed the risk of bias in including studies. Disagreement was resolved by consensus.

### 2.5. Statistical Analysis

A meta-analysis was conducted using Stata SE version 13.1 software (StataCorp, College Station, TX, USA). A random-effects model was employed to summarize the relationship between each SNP and preeclampsia susceptibility. The Higgins I^2^ metric was used to measure heterogeneity among the included studies, with an I^2^ value of 0% indicating no observed heterogeneity and a value above 50% indicating substantial heterogeneity. The strength of the association was assessed using adjusted odds ratios (ORs) and 95% confidence intervals (CIs). Pooled ORs were calculated using the allele genetic (additive), dominant, and recessive models. Funnel plots and Egger’s tests were performed to evaluate publication bias, with a significance level of 0.05.

## 3. Results

### 3.1. Characterization of Eligible Studies

**Figure 1:**
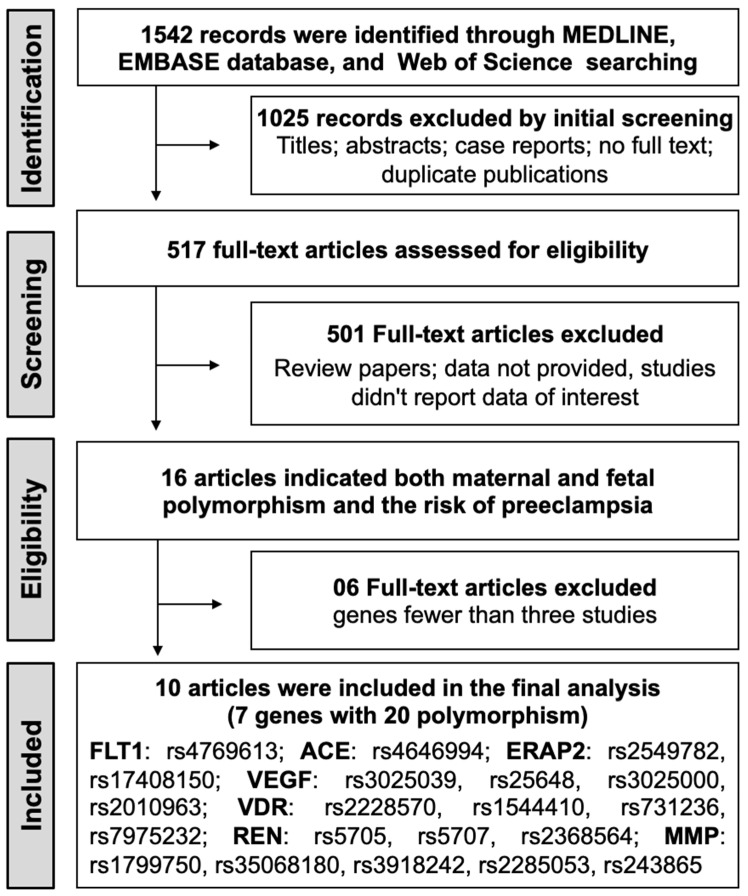
PRISMA flow chart of the study selection process for the meta-analysis.

The initial search identified 1542 potential records from electronic databases, and 517 full-text articles were evaluated for eligibility. Among these, 15 articles provided data on both maternal and fetal polymorphisms in relation to preeclampsia risk. Genes implicated in a minimum of three studies were selected, resulting in the exclusion of five articles. Consequently, 10 observational studies that satisfied the inclusion criteria were included in the meta-analysis [32,36,37,38,39,41,42,43,44,45]. The detailed steps of the literature selection process are shown in Figure 1.

The information presented in Table 1 is a summary of the characteristics of studies published independently over 22 years from 1999 to 2020. These studies investigated the relationship between 20 polymorphisms in 7 genes and the risk of preeclampsia in 16,025 maternal and fetal cases and 2,994,233 maternal and fetal controls.

**Figure 2:**
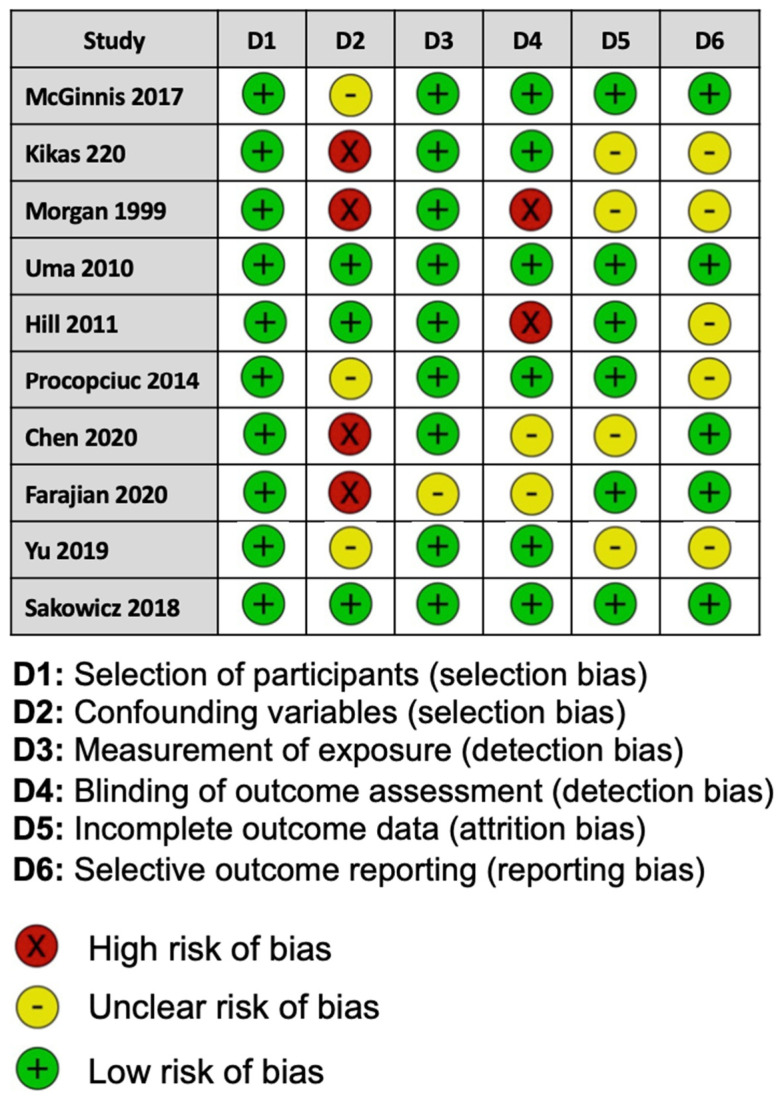
Assessment of risk of bias for each study in this meta-analysis using the Risk of Bias Assessment tool for Non-randomized Studies.

For the risk of bias for each study assessed, two of the ten studies included were considered to be at a low risk of bias, and three studies showed a high risk of bias. Five studies reported methods that raised concerns about the risk of bias (Figure 2).

### 3.2. Association between Maternal Genetic Variants and Preeclampsia

**Figure 3:**
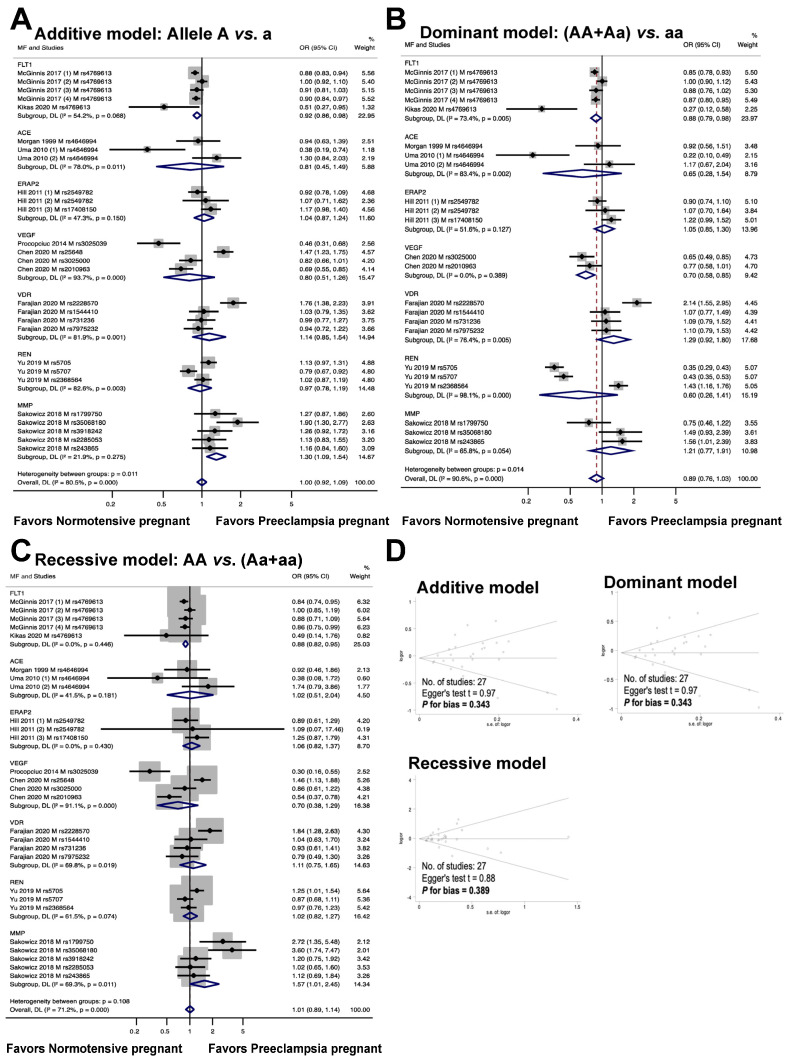
Association between maternal genetic variants and preeclampsia (**A**–**C**). Forest plot of maternal polymorphisms and preeclampsia in an additive model (**A**), dominant model (**B**), and recessive model (**C**). Funnel plot evaluating publication bias among studies included in the meta-analysis (**D**).

The relationship between maternal genetic variants and risk of developing PE is shown in Figure 3. Seven genes, including *FLT1*, *ACE*, *ERAP2*, *VEGF*, *VDR*, *REN*, and *MMP*, were analyzed for a total of twenty polymorphisms (*FLT1*: rs4769613; *ACE*: rs4646994; *ERAP2*: rs2549782, rs17408150; *VEGF*: rs3025039, rs25648, rs3025000, rs2010963; *VDR*: rs2228570, rs1544410, rs731236, rs7975232; *REN*: rs5705, rs5707, rs2368564; *MMP*: rs1799750, rs35068180, rs3918242, rs2285053, rs243865). The meta-analysis results showed that the maternal polymorphism *FLT1* rs4769613 was significantly associated with preeclampsia risk in the allelic model (additive model, OR 0.92, 95% CI 0.86 to 0.98, I^2^ = 54.2%, n = 5). Carriers of the dominant allele (AA and Aa) demonstrated a 12% reduction in preeclampsia susceptibility (dominant model, OR: 0.88, 95% CI 0.79 to 0.98, I^2^ = 73.4%, n = 5). The recessive model showed a significant association with preeclampsia risk (recessive model, OR 0.88, 95% CI 0.82 to 0.95, I^2^ = 0.0%, n = 5).

The association between the ACE rs4646994 polymorphism and the risk of preeclampsia is depicted in Figure 3. The results of the analysis of the three genotype models showed no statistically significant association between preeclampsia and maternal *ACE* rs4646994 polymorphism (additive model, OR 0.81, 95% CI 0.45 to 1.49, I^2^ = 78%, n = 3; dominant model, OR 0.65, 95% CI 0.28 to 1.54, I^2^ = 83.4%, n = 3; recessive model, OR 1.02, 95% CI 0.51 to 2.04, I^2^ = 41.5%, n = 3).

The association between the risk of preeclampsia and the maternal *ERAP2* rs2549782 and rs17408150 polymorphisms was assessed through a meta-analysis of three studies. The results are shown in Figure 3. The meta-analysis showed no statistically significant association (additive model, OR 1.04, 95% CI 0.87 to 1.24, I^2^ = 47.3%, n = 3; dominant model, OR 1.05, 95% CI 0.85 to 1.30, I^2^ = 51.6%, n = 3; recessive model, OR 1.06, 95% CI 0.82 to 1.37, I^2^ = 0.0%, n = 3) between the risk of preeclampsia and the maternal ERAP2 rs17408150 polymorphism.

The relationship between maternal *VEGF* polymorphisms (rs3025039, rs25648, rs3025000, and rs2010963) and preeclampsia risk is shown in Figure 3. In the dominant model, a significant association was observed between maternal VEGF polymorphisms and preeclampsia (OR 0.70, 95% CI, 0.58 to 0.85; I^2^ = 0.0%; n = 2), but no significant association was detected in the additive model (OR 0.80, 95% CI 0.51 to 1.26; I^2^ = 93.7%; n = 4) or recessive models (OR 0.70, 95% CI, 0.38 to 1.29; I^2^ = 91.1%; n = 4).

The meta-analysis depicted in Figure 3 demonstrated that the association between the risk of preeclampsia and maternal *VDR* polymorphisms (rs2228570, rs1544410, rs731236, and rs7975232) was not statistically significant (additive model, OR 1.14, 95% CI 0.85 to 1.54, I^2^ = 81.9%; n = 4; dominant model, OR 1.29, 95% CI 0.92 to 1.80, I^2^ = 76.4%, n = 4; recessive model, OR 1.11, 95% CI 0.75 to 1.65, I^2^ = 69.8%, n = 4).

The results of the meta-analysis depicted in Figure 3 indicated that there was no statistically significant connection between the risk of preeclampsia and maternal *REN* polymorphisms (rs5705, rs5707, and rs2368564) under the additive, dominant, or recessive models (OR 0.97, 95% CI 0.78 to 1.19, I^2^ = 82.6%, n = 3; OR 0.60, 95% CI 0.26 to 1.41, I^2^ = 98.1%, n = 3; OR 1.02, 95% CI 0.82 to 1.27, I^2^ = 61.5%, n = 3).

The association between maternal *MMP* genetic variations (rs1799750, rs35068180, rs3918242, rs2285053, and rs243865) and preeclampsia risk is shown in Figure 3. A meta-analysis revealed that the *MMP* variant was associated with an increased risk of preeclampsia in an allele-based genetic model (additive model: OR 1.30, 95% CI 1.09 to 1.54, I^2^ = 21.9%, n = 5). However, no significant association was observed between this genetic variant and the risk of preeclampsia in the dominant (OR 1.21, 95% CI, 0.77 to 1.91; I^2^ = 65.8%, n = 3) or recessive (OR 1.57, 95% CI 1.01 to 2.45, I^2^ = 69.3%, n = 5) models.

The graph in Figure 3D illustrates the publication bias in the meta-analysis. There was no evidence of publication bias in the studies, as shown by the symmetric shape of the Begg funnel plot (additive model, Egger’s test t = 0.97, P for bias = 0.343, n = 27; dominant model, Egger’s test t = 0.97, P for bias = 0.343, n = 27; recessive model, Egger’s test t = 0.88, P for bias = 0.389, n = 27).

### 3.3. Association between Fetal Genetic Variants and Preeclampsia

**Figure 4:**
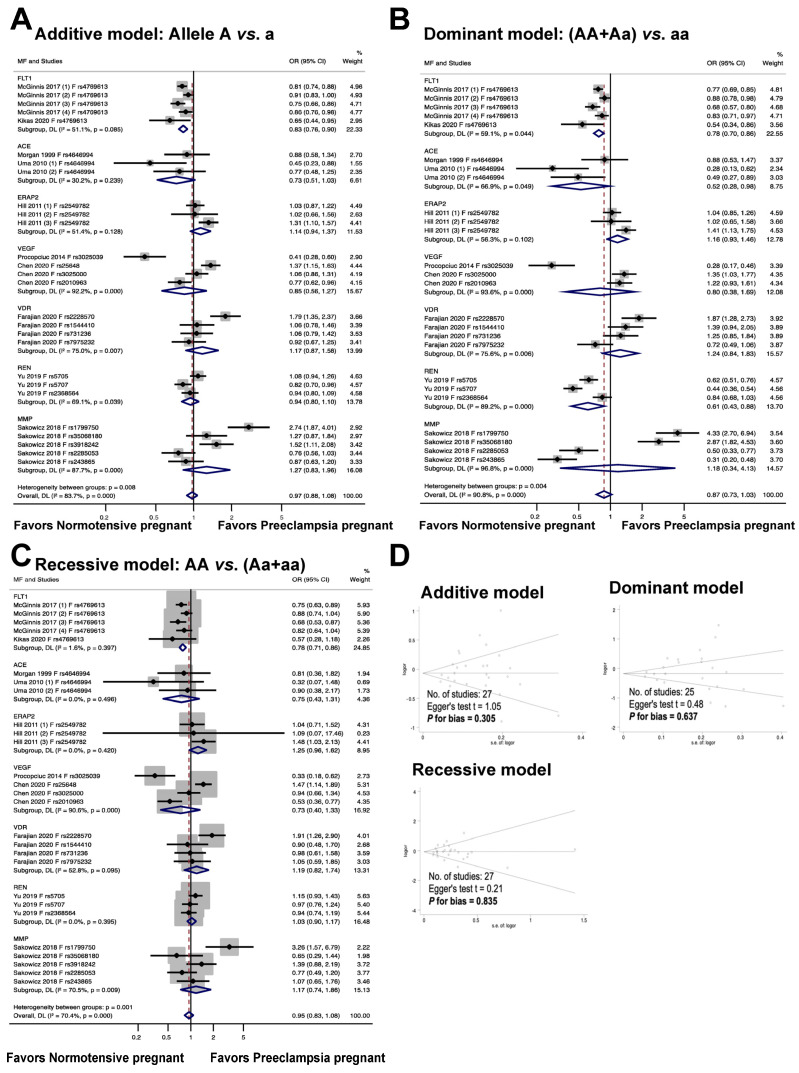
Association between fetal genetic variants and preeclampsia (**A**–**C**). Forest plot of maternal polymorphisms and preeclampsia in an additive model (**A**), dominant model (**B**), and recessive model (**C**). Funnel plot evaluating publication bias among studies included in the meta-analysis (**D**).

Figure 4 illustrates the association between fetal genetic variants and the risk of preeclampsia. The meta-analysis results reveal that the fetal polymorphism *FLT1* rs4769613 is significantly associated with an increased risk of preeclampsia in the allelic model, with an odds ratio of 0.83, a 95% confidence interval of 0.76 to 0.90, and an I^2^ of 51.1% (n = 5). In the dominant model, individuals with the dominant allele (AA and Aa) had a 22% increased risk of preeclampsia, with an odds ratio of 0.78, 95% confidence interval of 0.70 to 0.86, and I^2^ of 59.1% (n = 5). The recessive model also showed a significant association with preeclampsia risk, with an odds ratio of 0.78, 95% confidence interval of 0.71 to 0.86, and I^2^ of 1.6% (n = 5).

The findings from the meta-analysis revealed no statistically significant connection between the fetal *ACE* rs4646994 polymorphism and preeclampsia in the additive and recessive models. In the additive model, the odds ratio was 0.73, with a 95% confidence interval of 0.51 to 1.03 and an I^2^ of 30.2%. The recessive model showed an odds ratio of 0.75, 95% confidence interval of 0.43 to 1.31, and I^2^ of 0.0%. In contrast, a significant association was observed between fetal polymorphisms and preeclampsia under a dominant genetic model, with an odds ratio of 0.52, 95% confidence interval of 0.28 to 0.98, and I^2^ of 66.9%.

The findings from the meta-analysis revealed that there was no statistically significant connection between the risk of preeclampsia and the fetal *ERAP2* rs2549782 polymorphism (additive model, OR 1.14, 95% CI 0.94 to 1.37, I^2^ = 51.4%, n = 3; dominant model, OR 1.16, 95% CI 0.93 to 1.46, I^2^ = 56.3%, n = 3; recessive model, OR 1.25, 95% CI 0.96 to 1.62, I^2^ = 0.0%, n = 3).

The association between fetal VEGF polymorphisms (rs3025039, rs25648, rs3025000, and rs2010963) and preeclampsia is illustrated in Figure 4. In the additive, dominant, and recessive models, no significant association was observed between fetal *VEGF* polymorphisms and preeclampsia (OR 0.85, 95% CI 0.56 to 1.27, I^2^ = 92.2%, n = 4; OR 0.80, 95% CI 0.38 to 1.69, I^2^ = 93.6%, n = 3; OR 0.73, 95% CI 0.40 to 1.33, I^2^ = 90.6%, n = 4).

The association between the risk of preeclampsia and fetal *VDR* polymorphisms (rs2228570, rs1544410, rs731236, and rs7975232) is illustrated in Figure 4. The meta-analysis showed that the association between the risk of preeclampsia and fetal *VDR* polymorphisms was not statistically significant (additive model, OR 1.17, 95% CI 0.87 to 1.58; I^2^ = 75.0%; n = 4. dominant model, OR 1.24, 95% CI 0.84 to1.83; I^2^ = 75.6%, n = 4. recessive model, OR 1.19, 95% CI 0.82 to 1.74, I^2^ = 52.8%, n = 4).

The findings show that there was no statistically significant association between the risk of preeclampsia and fetal *REN* polymorphisms (rs5705, rs5707, and rs2368564) (additive model: OR 0.94, 95% CI 0.80 to 1.10, I^2^ = 69.1%, n = 3; dominant model: OR 0.61, 95% CI 0.43 to 0.88, I^2^ = 89.2%, n = 3; recessive model: OR 1.03, 95% CI 0.90 to 1.17, I^2^ = 0.0%, n = 3). 

The results of the meta-analysis indicated that the relationship between fetal *MMP* gene variations (rs1799750, rs35068180, rs3918242, rs2285053, and rs243865) and the likelihood of developing preeclampsia was not statistically significant across all models tested, including the additive (OR 1.27, 95% CI 0.83 to 1.96, I2 = 87.7%, n = 5), dominant (OR 1.18, 95% CI 0.34 to 4.13, I2 = 96.8%, n = 4), and recessive (OR 1.17, 95% CI 0.74 to 1.86, I2 = 70.5%, n = 5) models.

The meta-analysis illustrates the publication bias among the studies. It is important to note that there was no publication bias detected in the studies (as evidenced by the symmetric plot of the Begg funnel, and the results of the additive model, Egger’s test t = 1.05, P for bias = 0.305, n = 27; the dominant model, Egger’s test t = 0.48, P for bias = 0.637, n = 25; and the recessive model, Egger’s test t = 0.21, P for bias = 0.835, n = 27).

### 3.4. Association between Maternal and Fetal FLT1 Gene Regulatory Area rs4769613 Polymorphism and Preeclampsia

**Figure 5:**
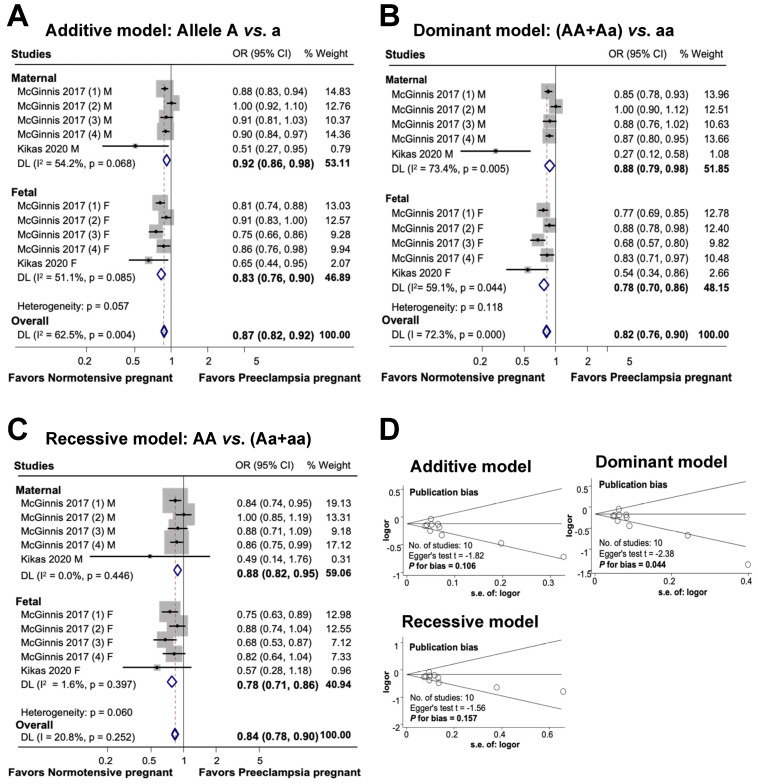
Association between maternal and fetal *FLT1* rs4769613 polymorphism and preeclampsia (**A**–**C**). Forest plot of maternal and fetal *FLT1* rs4769613 polymorphism and preeclampsia in additive (**A**), dominant (**B**), and recessive (**C**) models. Funnel plot evaluating publication bias among studies included in the meta-analysis (**D**).

The findings of this study on the association between maternal and fetal *FLT1* rs4769613 polymorphisms and preeclampsia are shown in Figure 5. In an additive model, a substantial association was observed between maternal polymorphisms and preeclampsia risk. Additionally, a significant association was discovered between genetic variants and diseases in the fetal additive model. Overall, the association between maternal and fetal polymorphisms and preeclampsia risk was substantial in the additive model (OR 0.87, 95% CI 0.82 to 0.92, I^2^ = 62.5%, n = 10). A significant association was observed between FLT1 rs4769613 and preeclampsia in both dominant and recessive models for mothers and fetuses. The overall significant association between maternal and fetal polymorphisms and preeclampsia was determined using a dominant model (OR 0.82, 95% CI 0.76 to 0.90; I^2^ = 72.3%; n = 10), which demonstrated an 18% reduction in susceptibility to the disease in individuals carrying one dominant allele. Finally, the association between maternal, fetal, and combined polymorphisms and preeclampsia was statistically significant in the recessive model (OR 0.84, 95% CI 0.78 to 0.90; I^2^ = 20.8%; n = 10).

The results of the additive and recessive models indicated no publication bias, as evidenced by the symmetric Begg funnel plot. Egger’s test for the additive model produced a value of t = −1.82, with a P for bias of 0.106 and n = 10. Similarly, Egger’s test for the recessive model yielded a value of t = −1.56, P for bias of 0.157, and n = 10. However, the dominant model showed evidence of publication bias, as indicated by Egger’s test, which produced a value of t = −2.38, P for bias of 0.044, and n = 10.

### 3.5. Association between Maternal and Fetal ACE rs4646994 Polymorphism and Preeclampsia

**Figure 6:**
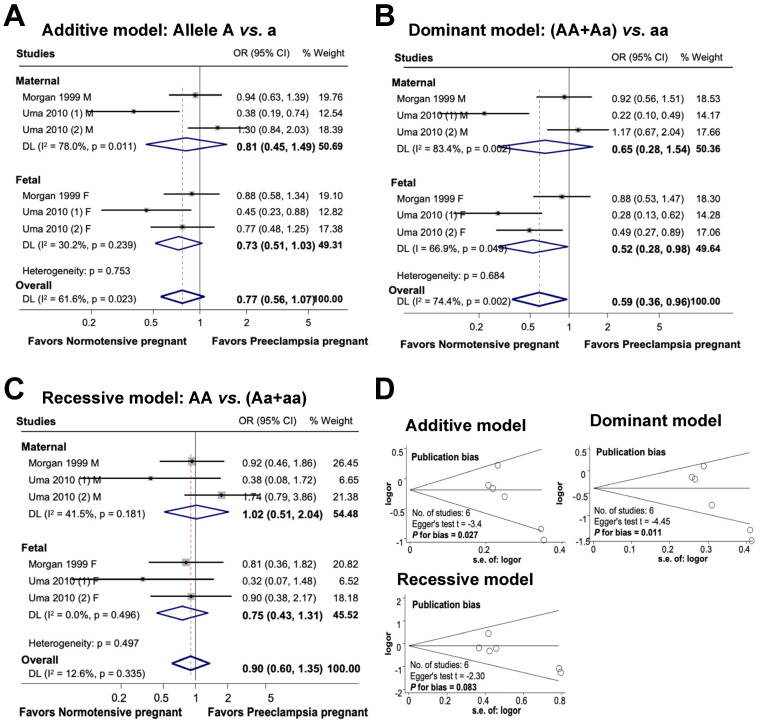
Association between maternal and fetal *ACE* rs4646994 polymorphisms and preeclampsia (**A**–**C**). Forest plot of maternal and fetal *ACE* rs4646994 polymorphisms and preeclampsia in an additive (**A**), dominant (**B**), and recessive (**C**) models. Funnel plot evaluating publication bias among studies included in the meta-analysis (**D**).

Figure 6 provides an overview of the association between the maternal and fetal *ACE* rs4646994 polymorphism and the risk of preeclampsia. In the additive model, there was no significant association between maternal genetic variations and the risk of preeclampsia or between fetal polymorphisms and preeclampsia. The combined influence of maternal and fetal polymorphisms on the risk of preeclampsia appeared to be significant under the additive model, with an odds ratio of 0.77 (95% CI 0.56 to 1.07; I^2^ = 61.6%; n = 6). In the dominant model, while there was no significant association between maternal polymorphisms and preeclampsia, the fetal ACE rs4646994 polymorphism was significantly associated with preeclampsia risk. Overall, the subjects carrying the dominant genotype had a 41% reduced risk of preeclampsia (OR 0.59, 95% CI, 0.36 to 0.96; I^2^ = 74.4%; n = 6). In the recessive model, no significant association was detected between maternal polymorphisms, fetal polymorphisms, and the risk of preeclampsia. The overall association between maternal and fetal polymorphisms and preeclampsia was not significant (OR 0.90, 95% CI 0.60 to 1.35; I^2^ = 12.6%; n = 6).

Publication bias was detected in both the additive and dominant models (additive model, Egger’s test t = −3.4, P for bias = 0.027, n = 6; dominant model, Egger’s test t = −4.45, P for bias = 0.011, n = 6). However, no publication bias was observed in the recessive model (Egger’s test t = −2.30, P for bias = 0.083, n = 6).

## 4. Discussion

The results of this study demonstrate that the FLT1 rs4769513 polymorphism in both the mother and fetus affects the likelihood of preeclampsia. In contrast, the ACE rs4646994 polymorphism in the fetus is associated with the risk of preeclampsia, whereas the ACE rs4646994 polymorphism in the mother is not. However, other polymorphisms did not display any substantial association with preeclampsia risk.

This meta-analysis integrated studies that concurrently examined both maternal and fetal genotypes and their association with preeclampsia. However, previous meta-analyses have only focused on studies that explore the association between maternal and fetal genotypes. Studies on individual gene polymorphisms are limited. Therefore, we advocate additional individual studies exploring the link between maternal and fetal gene polymorphisms and preeclampsia to complement the findings of this meta-analysis

A strong relationship is present between the mother and fetus throughout pregnancy and the placenta. The embryo obtains nourishment from its mother’s blood, and when the blood flows back, it contains various macromolecules such as proteins and miRNAs [46]. The process of the mother’s physiological response to this interaction is still being studied. However, the genetics of the fetus can also affect the health of the mother. A study of haplotype genetic scores in 10,734 mother–infant pairs of European ancestry revealed that alleles that raise fetal birth weight can also affect gestation duration and maternal blood pressure [47]. Additionally, research conducted by Rizzo et al. on a Hispanic population showed that the expression of fetal genes, regulated by DNA methylation, is associated with the risk of obesity and diabetes in the mother [48].

Preeclampsia is the most widely studied pregnancy-related disorder, and the advent of modern methods for obtaining fetal samples, such as amniocentesis and chorionic villus sampling, has provided scientists with greater opportunities to investigate fetal genetic material [49]. As discovered by Konecna et al., fetal RNA fragments in maternal blood can trigger an autoimmune response associated with preeclampsia [50]. Banadakoppa et al. found that certain haplotypes of fetal and maternal genetic variants can increase the risk of developing preeclampsia [51]. Steinthorsdottir et al. determined that preeclampsia results from a combination of maternal and fetal genetic materials and that late-onset preeclampsia tends to be linked to fetal genes [52].

The relationships between maternal and fetal variants on the development of preeclampsia (PE) is undeniable because *FLT1* plays a crucial role in placentation progression. Protein malfunction can result in vasoconstriction and PE [38,52,53,54]. *GCM1* and *TGF-β3* can affect maternal risk only if the mother carries the risk allele via fetal carriage [55,56]. However, some studies have revealed that fetal single-nucleotide polymorphisms (SNPs) may not significantly predispose mothers to PE [33,34,35]. The effects of maternal and fetal genes were investigated, including the interaction between fetal HLA-C2 and maternal KIR2DL1 on the KIRAA genotype [57] and the association of maternal and placental TABf haplotypes of the *VDR Taq1*, *Apa1*, *Bsm1*, and *Fok1* polymorphisms with a lower PE risk [43]. Additionally, paternal influence on PE risk was indicated through the *SOD2* Ala16Val SNP inherited from the father by the fetus [58].

An imbalance between pro- and anti-angiogenic factors is crucial for preeclampsia development. Elevated levels of sFlt-1, a splice variant of the *VEGF* endothelial receptor, have been observed in pregnant women with preeclampsia [59,60,61,62,63]. Many studies have shown that excessive circulating sFlt1 secreted by the placenta precedes the onset of preeclampsia and is correlated with disease severity [61,64,65,66,67]. sFlt-1 has a greater affinity for *VEGF* than cell-associated *VEGFRboactivities* and the balance of VEGF signaling in vascular development. sFlt1 inhibits the function of PIGF by inhibiting its interaction with intrinsic receptors, thereby diminishing the activities of VEGF and PIGF. Nonetheless, in cases of preeclampsia, the surplus release of this substance by the placenta disrupts the equilibrium between pro-angiogenic and anti-angiogenic elements, resulting in endothelial impairment, elevated blood pressure, and protein in the urine. The levels of sFlt-1 and the sFlt-1/PIGF ratio were found to be higher in preeclamptic patients than in the normal group, and can be used as markers for the differential diagnosis of preeclampsia [68]. The sFlt-1/PIGF ratio has been reported to have higher accuracy in differentiating preeclampsia patients from those without preeclampsia [69].

In *FLT1*, variant rs4769613, positioned on chromosome 13 (GRCh38.p14), has been linked to an increased risk of preeclampsia (PE) in the placenta. This variant, known as a single-nucleotide variant (SNV), modulates gene expression in the placenta and is referred to as the expression quantitative trait locus (eQTL). The *FLT1* rs4769613 variant (C > T) functions as a conditional eQTL, with the C allele thought to alter the regulation of the *FLT1* gene and enhance its expression. Studies have shown that placentas from women with the CC genotype have higher *FLT1* expression levels than placentas with the CT/TT genotype [70]. Previous studies have also identified the C/T variant rs4769613, located near FLT1, as a strong risk factor for preeclampsia [38,39,71]. McGinnis et al. (2017) were the first to provide evidence that alterations in the *FLT1* locus in the human fetal genome are associated with an increased risk of preeclampsia. This finding was replicated in multiple European cohorts and led to the identification of the lead risk SNP rs4769613 as a significant factor in preeclampsia. The association between rs4769613 genotype and late-onset preeclampsia has also been investigated [38]. Furthermore, the risk of PE related to *FLT1* variants has been suggested to be influenced by fetal gene expression, regardless of maternal or paternal origin [53].

During pregnancy, the renin–angiotensin system (RAS) plays a pivotal role in the regulation of the blood volume and blood pressure balance [72]. Angiotensin-converting enzyme 2 (ACE2) is a key component of this system as it transforms angiotensin (ANG) II to Ang-(1–7) and is associated with preeclampsia and pregnancy outcomes [73,74]. ACE2 exhibits protective effects in the heart, lungs, and kidneys, which are crucial for maintaining blood pressure homeostasis [75,76].

ACE2 is involved in balancing the activities of the heart, lungs, and kidneys, and its levels are important for maintaining blood pressure homeostasis. It cleaves Angiotensin I, forming Angiotensin II, which increases the level of plasminogen activator inhibitor-1 (PAI-1), a regulator of the fibrinolytic system, and normal development of pregnancy [77]. However, in preeclamptic women, ACE concentration is higher than that in normal pregnancies, leading to an imbalance in the Angiotensin II and Ang-(1–7) pathways, which is consistent with the development of hypertension [78]. Increased ACE activity causes abnormal placental circulation and angiogenesis, resulting in an increased risk of the disease. Variants of ACE have been reported to be associated with preeclampsia risk [74,79,80,81,82].

*ACE* is situated at locus 17q23.3, encompasses 26 exons and 25 introns, and encodes an angiotensin-converting enzyme. One of the most widely investigated and prevalent single-nucleotide polymorphisms (SNPs) discovered in *ACE* is the insertion/deletion (I/D) variation (rs4646994). The presence of the insertion (I) allele or deletion (D) allele of an Alu repeat sequence in intron 16 results in three genotypes: DD, II, and ID. ACE I/D gene polymorphism is correlated with plasma ACE activity, and the DD genotype of the I/D polymorphism in ACE is the most susceptible to the disease [83]. In contrast, heterozygote ID individuals have demonstrated intermediate concentrations of ACE in the plasma and tissues [84], and those with both the I allele (II) are associated with the lowest ACE levels, potentially reducing the risk of disease [85]. D-allele carriers have been reported to exhibit increased ACE concentrations, which significantly affect hypertension [84,86], and high ACE activity in the DD genotype of the *ACE* I/D polymorphism is associated with preeclampsia [87]. Furthermore, body mass index and oxidative damage have been suggested to contribute to the development of preeclampsia, alongside the polymorphism rs4646994 (González-Garrido et al. 2017). Additionally, *ACE* I/D polymorphism has been linked to severe proteinuria and renal dysfunction in preeclamptic patients [88].

Meta-analyses conducted across various studies have investigated the association between genetic polymorphisms and the risk of preeclampsia (PE), yielding mixed results. Although some polymorphisms have been linked to an increased risk of PE, others have shown no significant association. For instance, IL-10 polymorphism [89], RAAS [90], FOXP3 [91], thrombophilia [92], and NOS3 [93] were found to be associated with an increased risk of PE. Conversely, the MMP9-1562C > T polymorphism [94], TLR4 polymorphisms [29], and GST deletions [95] were not significantly associated with PE risk. Weicheng Duan and colleagues conducted a meta-analysis for two SNPs (rs3025039 and rs2010963) in the vascular endothelial growth factor. The findings revealed that the two VEGF gene polymorphisms were connected to an increased risk of PE. Nevertheless, insufficient studies for rs3025039 and rs2010963 SNP in fetal genetics prevented the current meta-analysis from drawing definitive conclusions about each SNP [18].

Shaik et al. conducted a meta-analysis of ACE gene polymorphisms and their correlation with preeclampsia risk. The results revealed no significant association between ACE polymorphisms and preeclampsia risk [96]. Similarly, the current meta-analysis found no significant association between the maternal ACE rs4646994 genetic variation and preeclampsia risk. However, the fetal ACE rs4646994 polymorphism was significantly associated with preeclampsia risk in a dominant model.

The heterogeneity index of several analyses in this study was acceptable. However, some analyses have revealed a relatively high heterogeneity index. The high heterogeneity between the studies is a limitation of the present study. Nonetheless, the results of the subgroup analysis, stratified by gene and maternal or fetal factors, showed that the heterogeneity index was not high for each subgroup analysis.

The current meta-analysis evaluated articles that examined the genotypes of both fetal and maternal genetic variations and preeclampsia. There are limited studies on fetal gene polymorphisms, which restrict the ability to categorize the analysis based on study design, publication year, etc. Although maternal and fetal genotypes may be related, the included studies did not consider this correlation, which could have influenced the outcomes of the meta-analysis.

## 5. Conclusions

In conclusion, a meta-analysis of 10 studies that assessed the association between 20 polymorphisms in 7 genes and the risk of preeclampsia in 16,025 maternal and fetal cases and 2,994,233 maternal and fetal controls found that maternal and fetal polymorphisms play a significant role in increasing the risk of preeclampsia, which could be used as a predictive factor. Future research should be conducted to understand the underlying mechanisms linking maternal and fetal polymorphisms to preeclampsia and provide guidance on screening for these polymorphisms in pregnant women.

## Figures and Tables

**Table 1:** Characteristics and Distribution of Studies Examining the Association between Maternal and Fetal Genetic Variants and Preeclampsia.

Gene ID	Variants	Ethic Group	Patient		Genotype Distribution						Allele Distribution			
			PE	Normal	PE			Normal			PE		Normal	
					AA	Aa	aa	AA	Aa	aa	A	a	A	a
***FLT1***														
***Maternal***														
McGinnis 2017 (1) M	rs4769613	UK	1875	5083	371	926	578	1152	2525	1396	1669	2081	4839	5327
McGinnis 2017 (2) M	rs4769613	Norwegian	1169	1927	270	584	315	444	962	521	1125	1213	1850	2004
McGinnis 2017 (3) M	rs4769613	Finnish	729	870	190	364	175	248	433	189	745	713	929	811
McGinnis 2017 (4) M	rs4769613	Icelandic	1205	135,190	236	595	374	29,737	67,335	38,118	1068	1342	126,808	143,572
Kikas 220 M	rs4769613	Estonian	18	135	3	9	6	39	80	16	15	21	158	112
***Fetal***														
McGinnis 2017 (1) F	rs4769613	UK	1004	5083	181	490	333	1152	2525	1396	851	1157	4839	5327
McGinnis 2017 (2) F	rs4769613	Norwegian	1125	1927	234	557	334	444	962	521	1026	1224	1850	2004
McGinnis 2017 (3) F	rs4769613	Finnish	527	870	112	262	153	248	433	189	487	567	929	811
McGinnis 2017 (4) F	rs4769613	Icelandic	411	135,190	77	202	132	29,737	67,335	38,118	356	466	126,808	143,572
Kikas 2020 F	rs4769613	Estonian	44	1724	9	22	13	535	870	319	40	48	1940	1508
***ACE***														
***Maternal***														
Morgan 1999 M	rs4646994	White Caucasian	72	83	18	31	23	22	36	25	67	77	80	86
Uma 2010 (1) M	rs4646994	Caucasian - early PE	22	105	2	8	12	22	61	22	12	32	105	105
Uma 2010 (2) M	rs4646994	Caucasian - late PE	38	105	12	19	7	22	61	22	43	33	105	105
***Fetal***														
Morgan 1999 F	rs4646994	White Caucasian	66	79	12	33	21	17	39	23	57	75	73	85
Uma 2010 (1) F	rs4646994	Caucasian - early PE	19	78	2	10	7	21	46	11	14	24	88	68
Uma 2010 (2) F	rs4646994	Caucasian - late PE	36	78	9	18	9	21	46	11	36	36	88	68
***ERAP2***														
***Maternal***														
Hill 2011 (1) M	rs2549782	Chilean	528	575	57	233	238	69	261	245	347	709	399	751
Hill 2011 (2) M	rs2549782	African American	424	412	1	44	483	1	45	529	46	1010	47	1103
Hill 2011 (3) M	rs17408150	Chilean	528	575	78	208	138	63	196	153	364	484	322	502
***Fetal***														
Hill 2011 (1) F	rs2549782	Chilean	528	575	58	235	235	61	253	261	351	705	375	775
Hill 2011 (2) F	rs2549782	African American	375	462	1	40	487	1	43	531	42	1014	45	1105
Hill 2011 (3) F	rs17408150	Chilean	528	575	71	184	120	63	215	184	326	424	341	583
***VEGF***														
***Maternal***														
Procopciuc 2014 M	rs3025039	Romanian	47	94	20	23	0	70	21	3	63	23	161	27
Chen 2020 M	rs25648	Han Chinese	208	336	182	26	0	278	56	2	390	26	612	60
Chen 2020 M	rs3025000	Han Chinese	185	304	67	82	36	121	142	41	216	154	384	224
Chen 2020 M	rs2010963	Han Chinese	181	286	54	93	34	126	117	43	201	161	369	203
***Fetal***														
Procopciuc 2014 F	rs3025039	Romanian	47	94	19	23	5	63	28	3	61	33	154	34
Chen 2020 F	rs25648	Han Chinese	208	336	184	23	1	282	54	0	391	25	618	54
Chen 2020 F	rs3025000	Han Chinese	185	304	68	90	27	116	131	57	226	144	363	245
Chen 2020 F	rs2010963	Han Chinese	181	286	52	105	24	124	117	45	209	153	365	207
***VDR***														
***Maternal***														
Farajian 2020 M	rs2228570	Iranian	152	160	106	38	8	89	54	17	250	54	232	88
Farajian 2020 M	rs1544410	Iranian	152	160	39	86	27	40	90	30	164	140	170	150
Farajian 2020 M	rs731236	Iranian	152	160	59	71	22	65	70	25	189	115	200	120
Farajian 2020 M	rs7975232	Iranian	152	160	36	95	21	45	91	24	167	137	181	139
***Fetal***														
Farajian 2020 F	rs2228570	Iranian	106	121	76	22	8	69	36	16	174	38	174	68
Farajian 2020 F	rs1544410	Iranian	106	121	21	69	16	26	71	24	111	101	123	119
Farajian 2020 F	rs731236	Iranian	106	121	45	45	16	52	47	22	135	77	151	91
Farajian 2020 F	rs7975232	Iranian	106	121	29	53	24	32	68	21	111	101	132	110
***REN***														
***Maternal***														
Yu 2019 M	rs5705	Han Chinese	272	608	226	41	5	485	119	4	493	51	1089	127
Yu 2019 M	rs5707	Han Chinese	276	648	136	107	33	342	270	36	379	173	954	342
Yu 2019 M	rs2368564	Han Chinese	262	598	157	96	9	363	206	29	410	114	932	264
***Fetal***														
Yu 2019 F	rs5705	Han Chinese	272	608	225	42	5	490	111	7	492	52	1091	125
Yu 2019 F	rs5707	Han Chinese	276	648	140	96	40	333	270	45	376	176	936	360
Yu 2019 F	rs2368564	Han Chinese	262	598	156	93	13	365	208	25	405	119	938	258
***MMP***														
***Maternal***														
Sakowicz 2018 M	rs1799750	Poland	86	85	30	22	34	14	43	28	82	90	71	99
Sakowicz 2018 M	rs35068180	Poland	86	85	32	31	23	12	43	30	95	77	67	103
Sakowicz 2018 M	rs3918242	Poland	86	85	61	25	0	57	26	2	147	25	140	30
Sakowicz 2018 M	rs2285053	Poland	86	85	67	19	0	66	17	2	153	19	149	21
Sakowicz 2018 M	rs243865	Poland	86	85	52	30	4	49	30	6	134	38	128	42
***Fetal***														
Sakowicz 2018 F	rs1799750	Poland	86	85	30	43	13	12	36	37	103	69	60	110
Sakowicz 2018 F	rs35068180	Poland	86	85	12	62	12	17	41	27	86	86	75	95
Sakowicz 2018 F	rs3918242	Poland	86	85	67	19	0	61	21	3	153	19	143	27
Sakowicz 2018 F	rs2285053	Poland	86	85	66	18	2	69	15	1	150	22	153	17
Sakowicz 2018 F	rs243865	Poland	86	85	50	27	9	48	34	3	127	45	130	40

## Data Availability

Data are contained within the article.
